# Sustainable intensification of crop residue exploitation for bioenergy: Opportunities and challenges

**DOI:** 10.1111/gcbb.12649

**Published:** 2019-10-31

**Authors:** Ioanna Mouratiadou, Tommaso Stella, Thomas Gaiser, Birka Wicke, Claas Nendel, Frank Ewert, Floor van der Hilst

**Affiliations:** ^1^ Copernicus Institute of Sustainable Development Utrecht University Utrecht The Netherlands; ^2^ Leibniz Centre for Agricultural Landscape Research (ZALF) Müncheberg Germany; ^3^ Institute of Crop Science and Resource Conservation University of Bonn Bonn Germany

**Keywords:** agricultural management scenarios, agricultural residues, biomass, climate change mitigation, greenhouse gas emissions, soil organic carbon, spatially explicit modelling, stakeholders, sustainable agricultural intensification, technical residue potentials

## Abstract

Crop residue exploitation for bioenergy can play an important role in climate change mitigation without jeopardizing food security, but it may be constrained by impacts on soil organic carbon (SOC) stocks, and market, logistic and conversion challenges. We explore opportunities to increase bioenergy potentials from residues while reducing environmental impacts, in line with sustainable intensification. Using the case study of North Rhine‐Westphalia in Germany, we employ a spatiotemporally explicit approach combined with stakeholder interviews. First, the interviews identify agronomic and environmental impacts due to the potential reduction in SOC as the most critical challenge associated with enhanced crop residue exploitation. Market and technological challenges and competition with other residue uses are also identified as significant barriers. Second, with the use of agroecosystem modelling and estimations of bioenergy potentials and greenhouse gas emissions till mid‐century, we evaluate the ability of agricultural management to tackle the identified agronomic and environmental challenges. Integrated site‐specific management based on (a) humus balancing, (b) optimized fertilization and (c) winter soil cover performs better than our reference scenario with respect to all investigated variables. At the regional level, we estimate (a) a 5% increase in technical residue potentials and displaced emissions from substituting fossil fuels by bioethanol, (b) an 8% decrease in SOC losses and associated emissions, (c) an 18% decrease in nitrous oxide emissions, (d) a 37% decrease in mineral fertilizer requirements and emissions from their production and (e) a 16% decrease in nitrate leaching. Results are spatially variable and, despite improvements induced by management, limited amounts of crop residues are exploitable for bioenergy in areas prone to SOC decline. In order to sustainably intensify crop residue exploitation for bioenergy and reconcile climate change mitigation with other sustainability objectives, such as those on soil and water quality, residue management needs to be designed in an integrated and site‐specific manner.

## Graphical abstract

Can we exploit crop residues for bioenergy production towards climate change mitigation without causing adverse impacts on the environment? We use stakeholder interviews, spatiotemporally explicit modelling and estimations of residue potentials and greenhouse gas (GHG) emissions to answer this question. Limited amounts of residues are exploitable in areas prone to soil organic carbon (SOC) decline. However, the adoption of integrated site‐specific management (SI) combining humus balancing (HB‐400), optimized fertilization (OptFert) and winter soil cover (FullCov) increases residue potentials while reducing SOC losses, GHG emissions and nitrate leaching. Therefore, it reconciles climate change mitigation with improved soil and water quality.

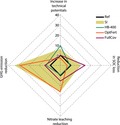

## 1 INTRODUCTION

Crop residues can play an important role in climate change mitigation in the coming decades, as an energy source with significant potential to substitute fossil fuels and contribute to greenhouse gas (GHG) emission reductions (Edenhofer et al., 2011). Unlike some other biomass feedstocks, their use has a low risk of causing direct and indirect land use change or other negative environmental and socio‐economic effects (Daioglou, Stehfest, Wicke, Faaij, & van Vuuren, 2016). In this context, second‐generation biofuels from crop residues appear as a favourable alternative to fossil transport fuels in Europe (Glithero, Ramsden, & Wilson, 2013; Gnansounou, 2010; Lindorfer, Fazeni, & Steinmüller, 2014). In particular, bioethanol from straw holds promise, as this material is the major contributor of residual biomass at the European level (Hamelin, Borzęcka, Kozak, & Pudełko, 2019). However, residue exploitation for bioenergy is subject to several barriers such as environmental concerns and market, logistic and biomass conversion challenges (IEA Bioenergy, 2017). One of the most acknowledged limitations to large‐scale crop residue exploitation is its likely impacts on long‐term soil functioning, productivity and associated ecosystem services due to reduction of the soil organic carbon (SOC) pool (Carvalho, Hudiburg, Franco, & DeLucia, 2017; Cherubin et al., 2018; Lal, 2005).

For climate change mitigation efforts not to compromise other sustainability objectives, it is important to increase residue harvests without adversely affecting overall agricultural productivity and environmental sustainability. Sustainable intensification (SI) of agricultural production can be a valuable framework to evaluate the opportunity of residue exploitation, due to its dual aim to increase productivity and environmental sustainability. Agricultural management measures aiming at SI, such as locally adapted practices for residue removal (IEA Bioenergy, 2017), possibly combined with targeted fertilization (Lindorfer et al., 2014) and cover crops (Liska et al., 2014), are often proposed in the literature as promising practices. Nonetheless, the potential of such measures to enhance the sustainability of bioenergy exploitation remains underexplored (Kluts, Wicke, Leemans, & Faaij, 2017; Liska et al., 2014).

These management measures could affect yields, environmental impacts and consequently sustainable residue extraction rates and emissions from both the land use and the energy sectors. All the above are associated with significant spatial variability (Haase, Rösch, & Ketzer, 2016; van der Hilst et al., 2010; 2012; Zhao et al., 2015), since they depend on the interaction of agricultural management with site‐specific pedoclimatic conditions and land use (Larsen, Bruun, & Lindedam, 2012; Monforti, Bódis, Scarlat, & Dallemand, 2013). Therefore, the need for detailed site‐specific modelling approaches for the assessment of sustainable biomass utilization for bioenergy is well emphasized in the literature (Brandão, Milà i Canals, & Clift, 2011; Monforti et al., 2013). Still, estimations of residue potentials are often based on static crop‐to‐residue ratios and sustainable removal rates set to regional averages. A limited number of studies use comprehensive modelling methodologies for the assessment of sustainable crop residue removal practices (e.g. Monforti et al., 2015; Zhao et al., 2015). Yet, even such studies typically evaluate residue removal in isolation to other management factors, and therefore fail to identify potentially synergistic effects of integrated agricultural management.

Beyond quantitative sustainability assessments, stakeholder perceptions on the prospects of bioenergy are of importance in the debate. Stakeholders can be a valuable source of critical information on successful agricultural and environmental policy formulation, implementation and decision‐making (Dwivedi & Alavalapati, 2009; Gregory & Wellman, 2001; Mouratiadou & Moran, 2007). Despite increasing attention to stakeholder perspectives in the field of bioenergy (Radics, Dasmohapatra, & Kelley, 2015), most quantitative studies still assess the potentials and sustainability of crop residue exploitation in isolation from local stakeholder perceptions.

Our study contributes to the debate on sustainable biomass production by combining a spatiotemporally explicit comprehensive assessment of agricultural management measures with stakeholder interviews to evaluate the prospects of sustainably intensifying crop residue exploitation for bioenergy. Using North Rhine‐Westphalia (NRW) in Germany as a case study, we first explore stakeholder views on barriers to sustainable crop residue exploitation. Next, we use agroecosystem modelling combined with estimations of energy potentials and GHG emissions to assess the performance of alternative agricultural management strategies via mid‐century projections. The synthesis of projected production and environmental effects with stakeholder views allows identifying in an integrated manner opportunities and challenges associated with the SI of crop residue exploitation for bioenergy.

## 2 MATERIALS AND METHODS

### 2.1 General methodological approach

**Figure 1:**
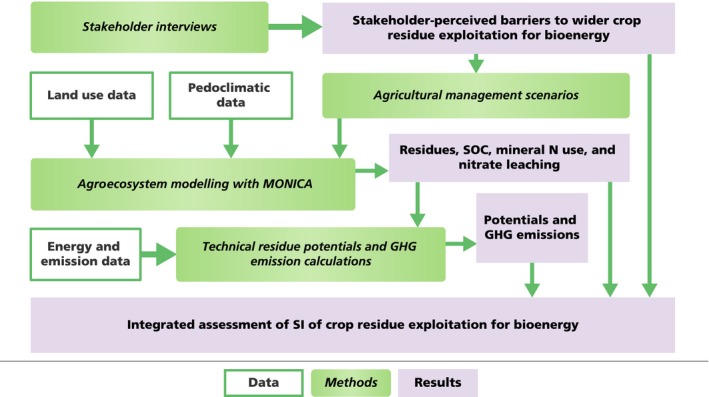
Overview of the data, methods and results of the study

Our methodology is based on the combination of stakeholder interviews and spatiotemporally explicit quantitative integrated assessment modelling applied within a scenario analysis (Figure 1). The study departed from interviews with local stakeholders to identify perceived barriers to the adoption of crop residues as a bioenergy feedstock in NRW (Section 2.2). The interviews identified impacts on soil fertility and yields as the most critical barrier. Therefore, in a second step, we developed agricultural management scenarios to explore opportunities to ameliorate these effects (Section 2.3.5). The scenarios assessed the measures of (a) residue management for straw and maize stover (e.g. balancing humus supply and demand), (b) optimizing mineral nitrogen (N) fertilization and (c) increasing the rate of soil winter cover crops, as well as (d) integrating all three measures. The scenarios were assessed via agroecosystem modelling using the MONICA model (MOdel for NItrogen and Carbon in Agroecosystems; Nendel et al., 2011; Section 2.3.1). MONICA provided spatiotemporally explicit scenario‐specific outputs over NRW in the period 1971–2060 on: extracted residues, residues remaining on the field, SOC in topsoil (30 cm), mineral N use and nitrate leaching. We combined MONICA outputs with energy and GHG emission data to estimate the effect of management on technical residue potentials and GHG emissions associated with the displacement of fossil fuels by bioethanol, changes in SOC, nitrous oxide emissions (N_2_O) and emissions from the production of the utilized fertilizers (Section 2.4). We analysed our results on technical residue potentials, SOC changes, nitrate leaching and GHG emissions (a) aggregated for the whole of NRW to identify regional impacts and (b) spatially explicit across NRW's landscape to explore their spatial variability. We combined the insights derived from the qualitative and quantitative assessments to draw a broader picture of the prospects of crop residue exploitation for bioenergy.

### 2.2 Stakeholder interviews

We conducted nine semi‐structured expert interviews in January 2017. The interviewed stakeholders included the representatives of public bodies (see interviews 1, 2 and 3 below), the farming sector (4, 5 and 6), environmental protection (7) and academia (8 and 9):

1. Ministry of Environment, Agriculture, Nature and Consumer Protection
2. State farmer advisory service Landwirtschaftskammer Nordrhein‐Westfalen (LWK NRW; 2 people)
3. State environmental protection agency LANUV (2 people)
4. Farmer association Westfälisch‐Lippischer Landwirtschaftsverband e.V. (1 person)
5. Farmer association Biokreis NRW (1 person)
6. A conventional farmer
7. Nature protection NGO NABU NRW (1 person)
8. A scientist of the University of Bonn with expertise on bioenergy crops
9. A scientist of the University of Bonn with expertise on local agricultural management practices

During the interviews, we asked stakeholders (a) via open‐ended discussion to express their opinion on the potential of crop residues as a bioenergy feedstock in NRW and (b) via a scoring exercise to characterize a set of potential barriers to the exploitation of crop residues for bioenergy as ‘unimportant’, ‘important’ or ‘very important’. These barriers comprised the following aspects:

- Agronomic and environmental aspects:
  ○ impacts on soil fertility and yields due to soil organic matter decline
  ○ increase of soil erosion
  ○ impacts on soil water functions (water infiltration, water holding capacity)
  ○ changes in fertilization requirements
  ○ farmers' experience in residue management
- Market and value chain:
  ○ competition with other uses (animal husbandry, horticulture)
  ○ insufficient market and technological development
  ○ inappropriate value‐chain networks
- Harvesting and logistics:
  ○ inappropriate harvesting equipment
  ○ insufficient storage capacity
  ○ bulky and costly transportation

The interviews allowed prioritizing the barriers likely to hinder large‐scale crop residue exploitation for bioenergy as perceived by the stakeholders. First, this guided our scenario design: with impacts on soil fertility and yields identified as the most important barrier (see Section 3.1) our scenarios focused on agricultural management measures to ameliorate these effects. Second, the evaluation of barriers by the stakeholders provided a wider outlook on other challenges towards crop residue exploitation for bioenergy.

### 2.3 Agroecosystem modelling and spatiotemporal aggregation

#### 2.3.1 Model choice and description

MONICA is a process‐based spatiotemporally explicit model which extends the crop model HERMES (Kersebaum & Richter, 1991) with the algorithms for the calculation of organic matter turnover of the DAISY model (Abrahamsen & Hansen, 2000), thus enabling the simulation of long‐term effects of agricultural management on SOC (e.g. Specka et al., 2016). Generic in its crop part, MONICA is designed to simulate different crops in sequence, addressing the carry‐over effects for soil water and nutrients in crop rotations (Kollas et al., 2015). MONICA has been intensively tested for simulating several of the main crops that appear in this study (Section 2.3.2; e.g. Asseng et al., 2013; Bassu et al., 2014; Fleisher et al., 2017; Kollas et al., 2015; Nendel et al., 2011; Rötter et al., 2012) and calibrated for high‐resolution simulations of wheat and maize in NRW (Hoffmann, Zhao, et al., 2016; Zhao et al., 2016).

#### 2.3.2 Pedoclimatic and land use data

For the MONICA simulations, we used simulated historical (1971–2005) and projected (2006–2050) bias‐corrected daily weather data provided by the Inter‐Sectoral Impact Model Intercomparison Project (ISIMIP2a data, Hempel, Frieler, Warszawski, Schewe, & Piontek, 2013). The projected data correspond to the Representative Concentration Pathway (RCP) 2.6 simulated by the IPSL‐CM5A‐LR Global Circulation Model (Dufresne et al., 2013). The RCP2.6 scenario was selected due to its milder climate signal compared to other RCPs and its proximity to climate targets stipulated by the Paris Agreement (UNFCCC, 2015).

Data on soil physical and chemical characteristics in NRW were retrieved from Hoffmann, Enders, Siebert, Gaiser, and Ewert (2016). The SOC values initially proposed in this database were modified such that they approximate the SOC levels proposed by Grosz et al. (2017), which have been shown to be closer to observed SOC contents on cropland. In order to ensure a consistent simulation of the SOC dynamics, data from long‐term field experiments carried out in Müncheberg, where alternative residue managements and organic fertilization levels are tested (Rogasik, Schroetter, Funder, Schnug, & Kurtinecz, 2004), were used for calibrating model parameters (Stella et al., 2019). The most influential parameters on the relative change of SOC in NRW during the projection period were identified via sensitivity analysis (Herman & Usher, 2017; Morris, 1991) and then calibrated using an automatized algorithm for optimization (DE‐MCz, Houska, Kraft, Chamorro‐Chavez, & Breuer, 2015). After calibration, the model achieved satisfactory performance in reproducing the time courses of SOC measured in the fields (Stella et al., 2019; Figure S5).

Land use data for 2007 (Information und Technik service of NRW, 2009) were combined with the nine different pedoclimatic zones of NRW (Roßberg, Michel, Graf, & Neukampf, 2007) by Burkhardt and Gaiser (2010). The exercise identified 26 combinations of crop rotations and pedoclimatic zones, as well as their frequency (Figure S3). The simulated rotations consider all main arable crops cultivated in NRW (winter wheat, winter barley, winter triticale, spring barley, grain maize, silage maize, winter rapeseed, sugar beet, potato). These rotations have been assigned to the arable area of NRW, using the ATKIS cropland mask (LANUV, 2009) aggregated to the grid resolution of the soil and climate data (1 km^2^). For simplicity, we assume static land use and exclude competition of arable land with pastureland, with the latter being excluded from the simulated agricultural area.

#### 2.3.3 Simulation set‐up

MONICA was set up to simulate agricultural production and N and carbon (C) dynamics in the agroecosystem for the 26 identified combinations of rotations and pedoclimatic zones. Simulations were realized at 1 km^2^ resolution grid cells covering the extent of the federal state of NRW. On each grid cell, we simulated independently the rotations pertaining to the pedoclimatic zone associated with this grid cell (Figure S3). Simulations were repeated multiple times by shifting the first crop in the rotation on each grid cell without modifying the crop sequence, in order to consider all possible combinations of crops and years (Teixeira, Brown, Sharp, Meenken, & Ewert, 2015). The MONICA results from these combinations were averaged per grid cell and rotation.

MONICA simulations were performed for 1971–2060 with a daily time step. The simulation period was split into a spin‐up (1971–2004) and a projection (2005–2060) period. The spin‐up allowed to determine the initial distribution of C among soil organic matter pools (Basso et al., 2011). For each grid cell and crop rotation, the calibration of initial (1971) SOC allowed obtaining SOC concentrations by the end of the spin‐up in line with those reported in the soil database used in this study. Each rotation was simulated continuously without re‐initialization of soil variables over the whole simulation period. Management during the spin‐up period was set according to our reference scenario assumptions (see Ref, Section 2.3.5), in order to reach a common starting point for the scenario assessment in 2005. During the projection period, management assumptions were varied according to the scenarios defined in Section 2.3.5.

#### 2.3.4 Spatiotemporal aggregation

In a subsequent step, MONICA results were temporally and spatially aggregated. Variables displaying marked inter‐annual variability, but no clear trends over time (residue production, mineral N use, nitrate leaching) were averaged over the period 2041–2060 for the characterization of mid‐century (2050) projections. The nonlinear time trend and the reduced inter‐annual variability of SOC suggested adopting a different approach for this variable, for which we evaluated changes over time (∆SOC) by comparing the change in simulated SOC from 2000–2004 (referred to as ‘base year’) to 2046–2050 (referred to as ‘2050’).

In order to account for the occurrence of different rotations on each of the grid cells, we computed weighted averages of the simulated variables per grid cell based on the shares of the rotations per pedoclimatic zone of NRW (Equation 1; Figure S3). This was performed for (a) average (over the crops of a rotation) residues (kg dry matter [DM] ha^−1^ year^−1^), (b) absolute (% kg C/kg soil) and relative (% year^−1^ change between 2050 and base year) SOC, (c) mineral N use (kg N ha^−1^ year^−1^) and (d) nitrate leaching (kg N ha^−1^ year^−1^).

(1) $${\text{WVar}}_{i}=\sum_{r}{\text{Fr}}_{i,r}\times {\text{Var}}_{i,r},$$

where *i* is the grid cell, *r* is the rotation, Fr*_i_*_,_ *_r_* is the fraction of occurrence of rotation on the pedoclimatic zone corresponding to the grid cell (1 > Fr*_i_*_,_ *_r_* > 0; Figure S3), Var*_i_*_,_ *_r_* is the value of variable per grid cell and rotation as simulated by MONICA and WVar*_i_* is the weighted average of variable accounting for shares of rotations per grid.

#### 2.3.5 Agricultural management scenarios

In total, we assess eight agricultural management scenarios combining assumptions on residue management, mineral N fertilization and winter soil cover (Table 1). Our reference scenario (Ref) uses reference assumptions representing current practices regarding all three management features (see the two following sections for details). Four residue management scenarios (R‐100, HB‐0, HB‐400, R‐0) explore impacts of altering residue management assumptions. Two scenarios explore the impact of changing assumptions on mineral N fertilization rates (OptFert) or winter cover crops (FullCov) respectively. Our last scenario (SI) provides alternatives to the reference assumptions regarding all three management dimensions, integrating a stringent humus balance (like scenario HB‐400), increased mineral N fertilization precision (like OptFert) and full winter cover (like FullCov).

**Table 1:** Agricultural management scenarios assessed in our study. Cells in grey indicate differences to the reference scenario (Ref)

Scenario	Residue management	Mineral N fertilization	Winter cover rate
Ref	Residue removal rate: 33%	Rule‐based	25%
R‐100	Residue removal rate: 100%	Rule‐based	25%
HB‐0	Humus balance: 0 H_eq_	Rule‐based	25%
HB‐400	Humus balance: 400 H_eq_	Rule‐based	25%
R‐0	Residue removal rate: 0%	Rule‐based	25%
OptFert	Residue removal rate: 33%	Optimized	25%
FullCov	Residue removal rate: 33%	Rule‐based	100%
SI	Humus balance: 400 H_eq_	Optimized	100%

#### 2.3.6 Residue management

Our residue management scenarios focus on cereal crop residue removal, and in specific straw and maize stover. The use of non‐cereal residues for energy purposes is limited and they are typically assumed to be kept on the field (Weiser et al., 2014). Irrespective of the scenario, silage maize stubbles (i.e. about 10% of the stem mass) and residues from sugar beet, potato, winter rapeseed and cover crops are assumed to remain on the field. Removal of cereal residues is scenario dependent.

We explore five residue management options for cereal residues, as also mentioned in Section 2.3.5. These options include both uniform residue removal rates, and humus balancing that accounts for humus supply and demand from various sources (i.e. humus demand per crop, humus supply by organic fertilization, cover crops and crop residues). For a detailed description of humus balance approaches, we refer the reader to Brock et al. (2013). Humus balance approaches can be applied for assessing the share of residues which can be used in a sustainable manner (Haase et al., 2016) and such an approach is currently recommended by the NRW farm advisory service (LWK NRW, 2015). However, the precision and stringency of the currently proposed humus balance remain highly debated (Brock et al., 2013; Kolbe, 2010; Lindorfer et al., 2014), indicating that the performance of alternatives merits further assessment.

The simulated residue management options are as follows:

- *Residue removal rate*: We formulate three options of uniform residue removal rates assuming removal of 0% (R‐0), 33% (Ref) and 100% (R‐100) of the produced cereal crop residues from the field. The 0% and 100% residue removal options are benchmarks to explore the possible range of variation. The 33% removal case is used as our reference assumption, based on a report of the LWK NRW (2014), proposed guidelines (Münch, 2008) and assumptions in other studies (e.g. Weiser et al., 2014). In MONICA, this is implemented by a rule specifying the uniform (across grid cells and cereal crops) scenario‐specific removal rate.
- *Humus balance*: We assess two humus balance variations. The first one is based on the currently recommended humus balance, where the balance between humus supply and demand is set to zero humus equivalents (H_eq_; HB‐0; LWK NRW, 2015). Given debates on the appropriateness of the currently recommended humus balance, we also assess a more stringent level, which requires a positive balance of 400 H_eq_ (HB‐400, SI). This level was chosen after testing different balance levels for their capacity to increase overall residue extraction while ameliorating average soil organic matter effects. In both humus balance variations, we assume that at least 25% of produced residues remain on the field due to harvesting equipment constraints. This is within the typical range, with the majority of studies assuming recovery rates between 60% and 80% of the produced residues (Weiser et al., 2014). The humus balance was calculated for each grid cell and cropping season using MONICA (see Section S2.1 for details).

#### 2.3.7 N fertilization and cover crops

In addition to residue removal practices, our scenarios explore alternative N fertilizer applications and the possibility to increase soil winter cover. Increased precision of mineral N applications is likely to reduce nitrate leaching without influencing crop and residue yields. This is particularly important in NRW, where nitrate pollution is a major concern as acknowledged both by literature (LANUV, 2014) and local stakeholders during the interviews.

Two N fertilization options were tested. In both cases, organic N is assumed to be applied to main crops before sowing at a rate based on the organic N balance of farms estimated at district (Landkreis) level (LWK NRW, 2014, table 27, p. 52; Figure S4). Mineral N is applied according to the following rules:

- *Rule‐based*: This option assumes that a target value of mineral N fertilizer is set according to current recommendations (LWK NRW, 2016) and soil N supply is estimated from rules based on the soil type, organic fertilization and crop sequence including the presence of cover crops (see Section S2.2; Ref, R‐0, R‐100, HB‐0, HB‐400, FullCov scenarios).
- *Optimized*: This option achieves higher precision of N fertilization by assuming that farmers are able to determine the exact mineral N content in their fields (e.g. by sampling and quick lab analysis), and consequently adjust fertilization rates in each field to meet the mineral N target in the rooted zone given the observed soil mineral N content (OptFert, SI). In model simulations, the latter is mimicked by the simulated content of soil mineral N.

In addition, we looked at an increase in winter cover as another measure to preserve SOC. This measure fits with the Greening of the Common Agricultural Policy (EC, 2013) and, as mentioned during the interviews, is increasingly adopted in NRW. The winter cover scenarios focus on the frequency of cover crops in the rotations. We assume their occurrence before a summer crop in either 25% (Ref, R‐0, R‐100, HB‐0, HB‐400, OptFert scenarios) or 100% (FullCov, SI) of the cases.

Further details on fertilization and cover crop assumptions and data are provided in Section S2.2.

### 2.4 Estimation of technical residue potentials and GHG emissions

The estimated technical residue potentials refer to potentials that are available under current production and technological limitations. As such, we consider water and nitrogen limitations in the crop growth within the MONICA simulations. Additionally, a 25% restriction on non‐harvestable residues is implemented in the scenarios with a humus balance approach (see Section 2.3.6). In the residue removal rate scenarios, such a constraint is not necessary since uniform residue removal rates are explicitly specified. The potentials are determined for residue biomass on a higher heating value basis, according to the values proposed by Haase et al. (2016), Batidzirai et al. (2016) and Di Blasi, Tanzi, and Lanzetta (1997).

(2) $${\text{TPot}}_{\text{NRW}}=10^{-9}\times \sum_{i}{\text{ExRes}}_{i}\times \text{HHV},$$

where *i* is the grid cell (km^2^), TPot_NRW_ is the technical residue potentials (PJ/year) at the level of NRW in 2050 and ExRes*_i_* is the extracted residues (kg DM/km^−2^ year^−1^) estimated by MONICA (see Section 2.3.5.1); HHV is the higher heating value of residues (MJ/kg DM).

The GHG emission calculation focuses on emissions affected by our management assumptions. We consider (a) emissions associated with the displacement of fossil fuels assuming the available residues are used for conversion to bioethanol, (b) emission equivalents of changes in SOC, (c) direct and indirect N_2_O emissions from mineral N application, residues, N mineralization associated with loss of soil organic matter, N volatilization and leaching and (d) emissions involved in the production of the utilized mineral N fertilizers.

For the calculation of emissions from the displacement of fossil fuels by bioethanol, we combined our estimates on technical residue potentials with the bioethanol and fossil fuel emission factors reported in the European Renewable Energy Directive 2018/2011 (EU RED II; EC, 2018). The bioethanol emission factor accounts for emissions for the collection, processing, transport and distribution of residues (EC, 2018).

(3) $${\text{DEm}}_{\text{NRW}}={\text{TPot}}_{\text{NRW}}\times \text{Eff}\times \left(\text{BioEF}-\text{FosEF}\right),$$

where DEm_NRW_ is displaced GHG emissions (kt CO_2‐eq_/year) at the level of NRW in 2050 from the substitution of fossil‐based transport fuels by bioethanol, Eff is efficiency of conversion of residues into bioethanol, BioEF is the bioethanol emission factor (g CO_2‐eq_/MJ) and FosEF is the fossil fuel emission factor (g CO_2‐eq_/MJ).

The estimation of emission equivalents due to changes in SOC utilize MONICA estimates of SOC, which reflect the difference between C added by residues and organic fertilizer minus C lost by soil respirations.

(4) $${\text{SEm}}_{\text{NRW}}=\sum_{i}\Delta {\text{SOC}}_{i}\times \text{SOCEF},$$

(5) $$\begin{array}{cc} & \Delta {\text{SOC}}_{i}\\ & \;=\left[\frac{\left({\text{AbsSOC}}_{2050,i}-{\text{AbsSOC}}_{2005,i}\right)}{100}\times \frac{\text{BDens}}{1000}\times \text{Vol}\right]/\text{Nyr}, \end{array}$$

where SEm_NRW_ is GHG emission equivalents (kt CO_2‐eq_/year) from average yearly changes in SOC over NRW in 2050, ΔSOC*_i_* is average yearly change in topsoil SOC between base year (2000–2004) and 2050 (2046–2050; kt C km^−2^ year^−1^), SOCEF is the conversion factor of SOC into emission equivalents (kg CO_2‐eq_/kg C‐CO_2_), AbsSOC_2005_ and AbsSOC_2050_ is average SOC (% kg C/kg soil) in base year and 2050, respectively, as estimated by MONICA, BDens is soil bulk density (t/m^3^), Vol is volume of topsoil (km^3^/km^2^) and Nyr is 46 years between base year and 2050.

For the calculation of N_2_O emissions, we used the guidelines of the Intergovernmental Panel on Climate Change (IPCC) for the Tier 1 methodology of N_2_O emissions on managed soils (IPCC, 2006). These include (a) direct N_2_O emissions from the soils to which N is applied/released, (b) indirect emissions from volatilization of ammonia and nitrogen oxides, and their subsequent redeposition and that of their products to soils and waters and (c) indirect emissions after leaching of N from managed soils. We have not computed emissions that are not influenced by our scenario assumptions. These are emissions from organic N applications, urine and dung N deposition by grazing animals, N in below‐ground biomass other than for cover crops and N mineralization associated with loss of soil organic matter from change of land use.

For direct N_2_ Oemissions from managed soils we consider the emission sources of mineral N fertilizers, above‐ground residues that remain on the field, below‐ and above‐ground biomass of cover crops and N mineralization associated with loss of soil organic matter from change of management:

(6) $$\begin{array}{cc} {\text{DiN}}_{2}{\text{OEm}}_{\text{NRW}} & =\sum \left[{\text{Fert}}_{i}+\sum_{c}{\text{RetRes}}_{c,i}\times {\text{NRes}}_{c}\right.\\ & \;\;{\;+\;\text{AbCovCr}}_{i}\times \left(\text{NAbCov}+\text{FrAbBe}\times \text{NBeCov}\right)\\ & \;\;\left.-\Delta {\text{SOC}}_{i}\times \text{FrCN}\right]\times \text{FrND}\times \text{NiEF}\times N_{2}\text{OEF}, \end{array}$$

where c is crop, DiN_2_OEm_NRW_ is direct N_2_O GHG emission equivalents (kt CO_2‐eq_/year) from sources described above over NRW in 2050, Fert*_i_* is mineral N use (kt N/km^−2^ year^−1^), RetRes_c,_ *_i_* is above‐ground residues remaining on the field (kt DM/km^−2^ year^−1^), NRes_c_ is crop‐specific N concentration in above‐ground residues (kg N/kg DM), AbCovCr*_i_* is above‐ground cover crop biomass (kg DM/km^−2^ year^−1^), NAbCov is N concentration in above‐ground cover crop biomass (kg N/kg DM), FrAbBe is ratio of below‐ground to above‐ground cover crop biomass, NBeCov is N concentration in below‐ground cover crop biomass (kg N/kg DM), FrCN is N to C ratio (kg N/kg C), FrND is N emission factor from applications on managed soils (kg N‐N_2_O/kg N), NiEF is conversion factor of N‐N_2_O into N_2_O (kg N_2_O/kg N‐N_2_O) and N_2_OEF is global warming potential for N_2_O emissions (g CO_2‐eq_/g N_2_O). RetRes_c,_ *_i_*, Fert*_i_* and AbCovCr*_i_* are estimated by MONICA as described in Sections 2.3.6 and 2.3.7.

For indirect emissions, we consider N volatilization from mineral N applications and emissions from leaching:

(7) $$\begin{array}{cc} {\text{InN}}_{2}{\text{OEm}}_{\text{NRW}} & =\sum_{i}\left({\text{Fert}}_{i}\times \text{VoEF}+{\text{Leach}}_{i}\times \text{LeEF}\right)\\ & \;\;\times \text{NEF}\times N_{2}\text{OEF}, \end{array}$$

where InN_2_OEm_NRW_ is indirect N_2_O GHG emission equivalents (kt CO_2‐eq_/year) from volatilization, deposition and leaching over NRW in 2050; VoEF is emission factor of N volatilization and deposition (kg N‐N_2_O/kg N); Leach*_i_* is nitrate leaching (kt N/km^−2^ year^−1^) estimated by MONICA; LeEF is N emission factor from leaching (kg N‐N_2_O/kg N).

Emissions from the production of the utilized fertilizer are calculated in function of mineral N applied, as estimated by MONICA (see Section 2.3.7).

(8) $${\text{FEm}}_{\text{NRW}}=\sum_{i}{\text{Fert}}_{i}\times \frac{\text{FertEF}}{1000},$$

where FEm_NRW_ is GHG emissions from production of utilized mineral N fertilizer (kt CO_2‐eq_); FertEF is N fertilizer emission factor (g CO_2‐eq_/g N).

The utilized data are shown in Table 2.

**Table 2:** Data and assumptions for the estimation of technical potentials and greenhouse gas emissions

Parameter	Value	Source	Additional description
Higher heating value of residues (HHV)	17 MJ/kg DM	Batidzirai et al. (2016), Di Blasi et al. (2016), Haase et al. (2017)	In the range of values proposed by the three sources
Conversion efficiency of residues into bioethanol (Eff)	0.326	Lindorfer et al. (2012)	According to value proposed for straw; we assume the same value for maize stover
Soil bulk density (BDens)	1.4 t/m^3^	Hoffmann, Zhao, et al. (2016)	
Topsoil volume (Vol)	300 km^3^/km^2^	Own estimation	Volume accounting for 30 cm of topsoil.
Bioethanol emission factor (BioEF)	15.7 g CO_2‐eq_/MJ	EU RED II (EC, 2013)	Default value for wheat straw ethanol assumed to apply to all residues
Fossil fuel emission factor (FosEF)	94 g CO_2‐eq_/MJ	EU RED II (EC, 2013)	Fossil fuel comparator for transport biofuels
N_2_O global warming potential (N_2_OEF)	265 g CO_2‐eq_/g N_2_O	IPCC AR5 (Myhre et al., 2013)	
Conversion factor of soil C into CO_2_ emission equivalents (SOCEF)	3.67 (44/12) kg CO_2‐eq_/kg C‐CO_2_	IPCC (2006)	
Conversion factor of N‐N_2_O into N_2_O emissions (NiEF)	1.57 (44/28) kg N_2_O/kg N‐N_2_O	IPCC (2006)	
N emission factor from applications on managed soils (FrND)	0.01 kg N‐N_2_O/kg N applied	IPCC (2006)	
N volatilisation and deposition emission factor (VoEF)	0.10 × 0.01 kg N‐N_2_O/kg N	IPCC (2006)	
N leaching emission factor (LeEF)	0.0075 kg N‐N_2_O/kg N	IPCC (2006)	
N fertiliser production emission factor (FertEF)	5.89 g CO_2‐eq_/g N	BioGrace (2015)	
Crop‐specific N concentration in above‐ground residues (NRes_c_)	see Table S5	IPCC (2006)	Differentiated per crop
N concentration in above‐ground cover crop biomass (NAbCov)	0.015 kg N/kg DM	IPCC (2006)	Value for non‐N fixing forages
N concentration in below‐ground cover crop biomass (NBeCov)	0.012 kg N/kg DM	IPCC (2006)	Value for non‐N fixing forages
Below‐ to above‐ground cover crop biomass ratio (FrAbBe)	0.54	IPCC (2006)	Value for non‐N fixing forages
N to C ratio (FrCN)	0.1 kg N/kg C	IPCC (2006)	Default value for management changes on ‘Cropland Remaining Cropland’

## 3 RESULTS

### 3.1 Stakeholder perceptions on crop residues

The interviewed stakeholders perceived crop residues as an agricultural feedstock with limited potential for bioenergy production in NRW. Via open‐ended discussion, they identified the following barriers:

1. Impacts on soil fertility and yields due to a decline in soil organic matter (mentioned in nine interviews);
2. Competition with other uses such as animal bedding and horticulture (six interviews);
3. Insufficient technological developments for the conversion of residues into energy (three interviews);
4. Insufficient market developments with costs being disproportional to the expected fuel price (two interviews);
5. Logistic requirements regarding storage and transport (one interview).

**Figure 2:**
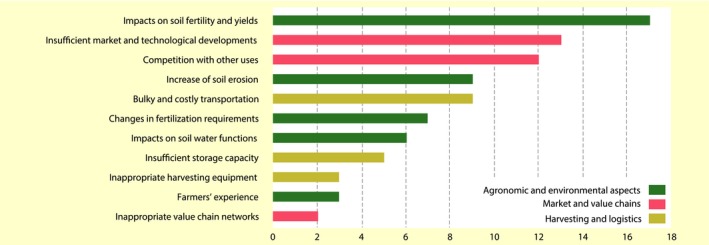
Scores of barriers to crop residue exploitation for bioenergy according to the interviewed stakeholders. For each interview, we attributed zero points to barriers perceived ‘unimportant’, one to those perceived ‘important’ and two for those seen as ‘very important’. We summed the points from all interviews in order to get the aggregate score per barrier

The results of the scoring exercise are consistent with those of the open‐ended discussion (Figure 2). Impacts on soil fertility and yields attracted most points. Competition with other uses, as well as insufficient development of technology and markets are following. Other limitations perceived as potentially important include an increase in soil erosion, impacts on soil water functions, concerns about logistic practicality and costs and adjustments in fertilization patterns which may lead to increased nitrate leaching. Storage capacity, harvesting equipment, farmers' experience and value chain networks were evaluated as unimportant challenges to the crop residue exploitation for bioenergy in NRW.

### 3.2 Technical residue potentials

**Figure 3:**
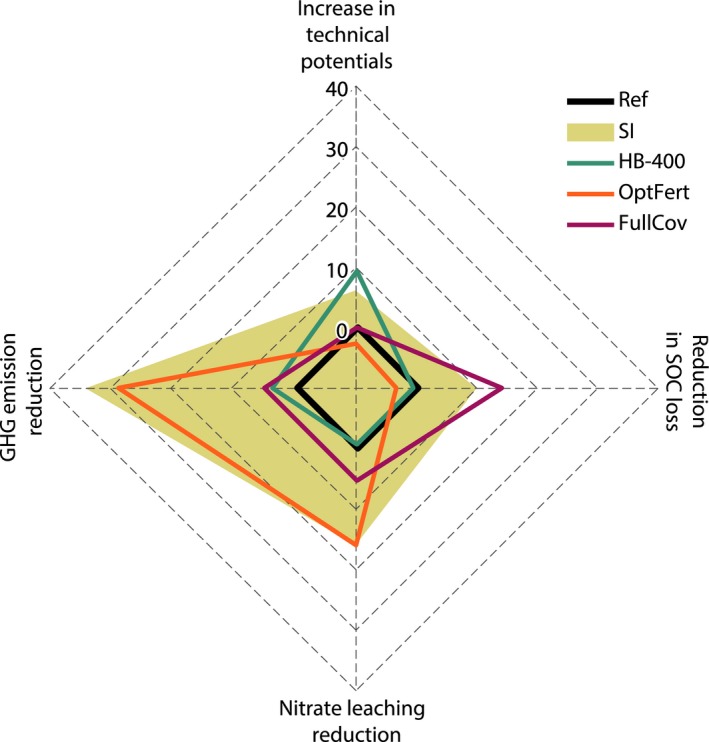
Change in technical potentials and environmental impacts between our reference scenario (Ref) and other agricultural management scenarios (SI, HB‐400, OptFert, FullCov) in 2050 (%). For technical potentials, a positive change represents an increase. For soil organic carbon (SOC), nitrate leaching and greenhouse gas (GHG) emissions, a positive change represents a decrease. The graph is based on the values shown in Table S6, estimated according to the equations shown in Section 2.4. We do not show the results of the benchmark R‐0, R‐100 and HB‐0 scenarios, in order to enhance the readability of the graph with respect to the results of the other five scenarios

Technical residue potentials in 2050 increase from 48 PJ/year in the Ref scenario to 50 PJ/year in the SI scenario (Figure 3; Table S6). This 5% increase is due to the combined effects of a stringent humus balance (HB‐400 scenario) and optimizing fertilization to meet the mineral N target in the rooted zone (OptFert scenario). HB‐400 allows for 9% higher aggregate residue extraction than Ref. In contrast, OptFert leads to a 2% decline in extracted residues and corresponding potentials, because it reduces fertilizer inputs significantly resulting in some small negative effects on residue yields. The full winter cover scenario (FullCov) has almost no impact on residue extraction. The scenario with zero residue extraction (R‐0) is associated with no technical residue potentials. On the other end of the spectrum, in scenarios with extremely high residue extraction (HB‐0 and R‐100) potentials are more than double or even triple than those in Ref respectively.

**Figure 4:**
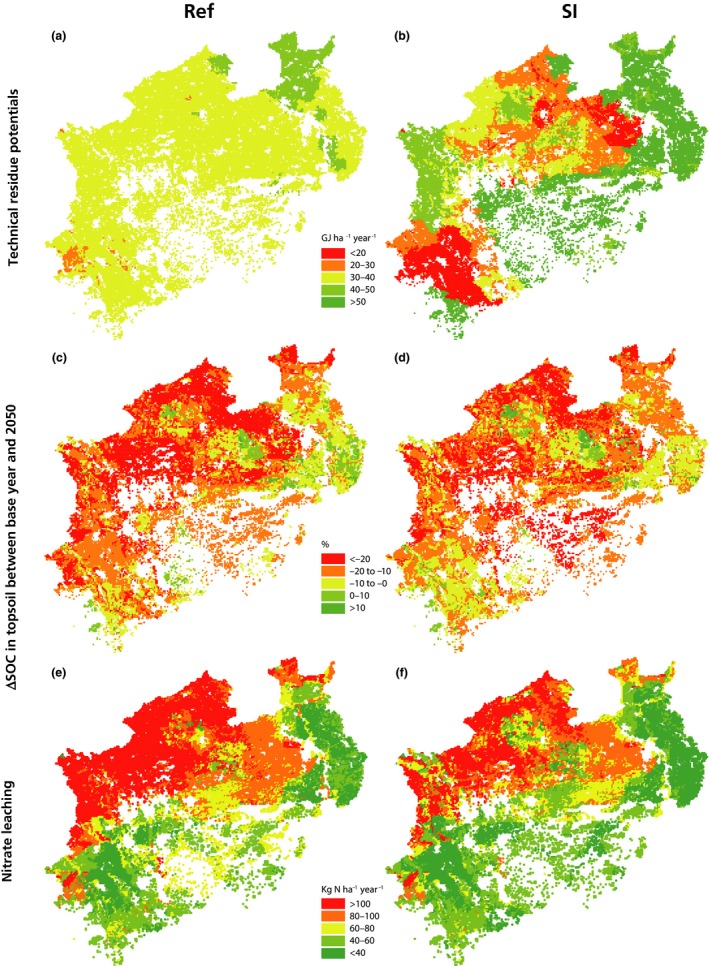
Spatially explicit technical residue potentials in 2050 (a, b), ∆SOC in topsoil between base year and 2050 (c, d) and nitrate leaching in 2050 (e, f) for the Ref and SI scenarios

Land use and organic fertilization patterns give rise to spatially variable results regarding the performance of the SI scenario compared to Ref (Figure 4a,b). In the Ref scenario, even though rotations and yields are spatially variable, technical potentials correspond to 30–40 GJ ha^−1^ year^−1^ in most of NRW's arable land, since we assume uniform residue extraction rates. In the SI scenario, where residue management is controlled by the humus balance approach, we notice that energy potentials are (a) <30 GJ ha^−1^ year^−1^ in much of the south west and central north parts of NRW, (b) 30–50 GJ ha^−1^ year^−1^ in the north‐west and (c) more than 50 GJ ha^−1^ year^−1^ in the north‐east and central south. Lower energy potentials occur in pedoclimatic regions with rotations with a high share of silage maize, sugar beet and potatoes (see Figure S3 for land use patterns). Theses rotations correspond to lower residue extraction. Sugar beet and potatoes have higher humus demand than other crops (see Table S1 for humus demand) while silage maize has lower humus supply (only stubbles remain on the field, as described in Section 2.3.6). Therefore, in rotations with these crops, higher residue retention from winter cereals is required to compensate for their higher humus demand and lower humus supply (see HUMCO in Equation 1 in Section S2.1). On the contrary, potentials are higher in areas where winter cereals are more dominant. Organic fertilization levels also play a role. For example, in the north west of the region, even though silage maize, sugar beet and potatoes are also considerably present in the rotational combinations, higher organic fertilization (see Figure S4 for organic fertilization patterns) allows for higher residue extraction.

### 3.3 SOC

Soil organic carbon declines over time irrespective of scenario and its agricultural management. This comes as no surprise in regions like NRW due to intensive cropping and management (Steinmann et al., 2016). In the Ref scenario, we simulate a loss of 281 kt of SOC in average per year in the period from the base year to 2050 from the arable land of NRW (Table S6). In the SI scenario, average annual SOC losses are 8% lower than in the Ref scenario (Figure 3). The main driver of this effect is the increase in winter soil cover. The FullCov scenario results in 14% lower SOC losses than the Ref scenario. The reduction in applied mineral N in the OptFert scenario and the humus balance in the HB‐400 scenario cause slightly higher SOC losses than Ref does (3% and 1% respectively). A combination of all three management options results in the differences between Ref and SI. Not surprisingly, keeping all residues on the ground (R‐0 scenario) is the most favourable scenario with respect to SOC. While SOC still declines, this decline is almost half of that observed in the Ref scenario. Conversely, the scenarios with high residue extraction (HB‐0 and R‐100) perform worse than all other scenarios. SOC losses are 52% and 88% higher than the Ref scenario respectively.

Distinct spatial patterns are observed with respect to SOC (Figure 4c,d; Figure S6), due to differences in soils and rotations. In both Ref and SI scenarios, SOC decline rates are higher than 20% compared to the base year period (2000–2004) in locations with light soils in the north of NRW (see Figure S2 for soil textures). In most other localities, decline rates range between 0% and 20%. In specific locations in the north, an increase over time in the order of 10% is observed, typically in areas with heavy soils and a low initial SOC budget, identified as the optimal conditions for halting SOC decline (Stella et al., 2019). In the SI scenario, SOC decline rates are lower than the Ref scenario in most of the region, since winter soil cover is higher and residue removal is tailored to land use and management. However, in the eastern and central southern parts of NRW, decline rates are higher than in Ref. In these areas, rotations with winter rapeseed are more dominant. These rotations seem to be performing worse in the SI scenario, indicating some discrepancy between the contribution of this crop to SOC assumed by the humus balance methodology and the modelling. The improvements induced by an increase in winter cover in the SI scenario are also less pronounced here, since summer crops do not make up a significant share of the assumed rotations.

### 3.4 Relationship between technical potentials and SOC

**Figure 5:**
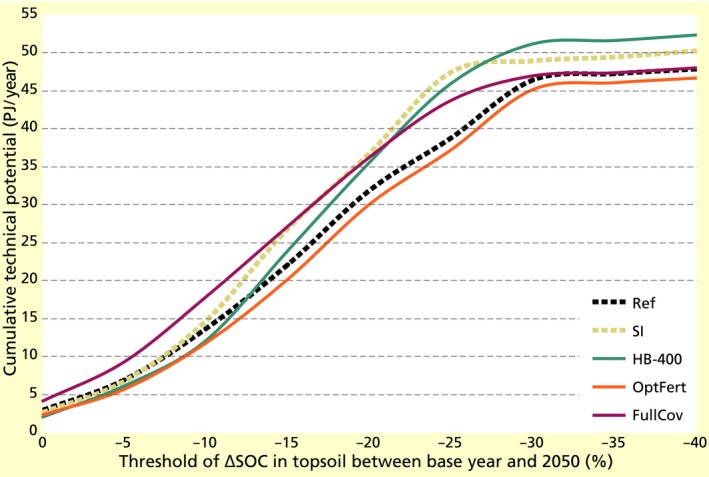
Cumulative technical residue potentials across North Rhine‐Westphalia in 2050 (PJ/year) at different thresholds of ∆SOC in topsoil between base year and 2050 (%) for the Ref, SI, HB‐400, OptFert and FullCov scenarios. Cumulative technical potentials are computed as the summation of potentials in grid cells where ΔSOC corresponds to values above a given threshold

Combining our results on technical potentials and SOC, we identify the grid cells below different thresholds of ∆SOC and estimate cumulative residue potentials corresponding to these cells (Figure 5). In a case with no SOC decline between the base year and 2050, only 2–4 PJ/year of residue potentials are exploitable. With a SOC decline of up to 15%, about half of the technical residue potentials of NRW (ca. 20–30 PJ/year depending on the scenario) can be utilized. Exploiting the other half, leads to SOC decline rates of up to 40%, although more than 95% of potentials correspond to decline rates below 25% (SI) or 30% (Ref, HB‐400, OptFert, FullCov).

At all thresholds of SOC decline, the SI scenario corresponds to residue potentials higher than or equal to those observed for the Ref scenario. At thresholds below 15%, the FullCov scenario is associated with the highest potentials compared to all other scenarios, due to the positive contribution of cover crops to SOC. The OptFert scenario corresponds to the lowest potentials at all levels of SOC decline, since the yield reductions occurring in this scenario result in fewer harvested but also fewer incorporated residues that would contribute to SOC stocks. The HB‐400 scenario also performs worse than Ref at these SOC levels, since it may lower SOC if used in isolation of other measures such as the use of cover crops. The above findings demonstrate that the ranking of different scenarios in terms of resulting exploitable residues, and consequently the optimal management strategy, varies depending on policy targets for SOC conservation.

### 3.5 Nitrate leaching

Also nitrate leaching is affected positively in the SI scenario (Figure 3). A 16% reduction is achieved compared to Ref (91 and 108 kt N/year, respectively; Table S6). The main driver is the reduction in mineral N fertilization rates, as similar reduction rates are observed in the OptFert scenario. Full winter cover (FullCov) also reduces nitrate leaching (5% compared to Ref), because it is related to slightly lower mineral N applications (see Section S2.2 in the *rule‐based* option N applications are adjusted for cover crops) and the uptake of N by the cover crops during winter. We observe a tendency for higher nitrate leaching (12% compared to Ref) in the scenario with zero residue removal (R‐0). The retention of more residues implies that more N is available for mineralization in the field, resulting in higher N surplus and risk of leaching. In contrast, leaching is reduced by 10% and 16%, respectively, in the scenarios with the zero humus balance (HB‐0) and maximal residue removal (R‐100), where less residues are left on the field. Nitrate leaching is considerably higher in the north‐west of NRW where light soils prevail and organic fertilization rates are higher (Figure 4e,f).

### 3.6 GHG emissions

**Figure 6:**
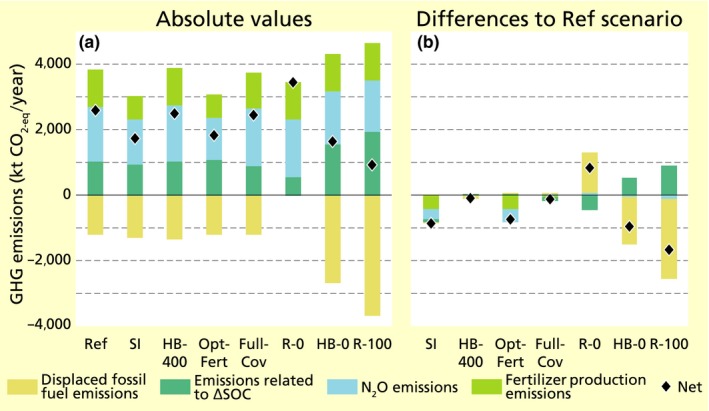
Greenhouse gas (GHG) emissions from the displacement of fossil fuels by bioethanol, changes in soil organic carbon, N_2_O emissions and production of fertilizers (kt CO_2_‐eq/year) in 2050. We show absolute values per scenario (a) and difference to the Ref scenario (b)

Greenhouse gas emissions associated with the four emission sources investigated in our study (substitution of fossil fuels by bioethanol, changes in SOC, N_2_O emissions, mineral N production) decline by 33% in the SI scenario compared to the Ref scenario (1,752 and 2,623 kt CO_2‐eq_/year, respectively; Figure 3; Table S6). All emission sources contribute to this: (a) displaced emissions from the substitution of fossil fuels increase by 5% due to higher technical residue potentials, (b) emission equivalents related to SOC changes drop by 8% due to lower SOC losses, (c) N_2_O emissions drop by 18% mainly due to lower mineral N fertilization and (d) emissions from the production of mineral N decline by 37% due to lower mineral N demand (Figure 6a,b; Table S7).

Greenhouse gas emissions drop significantly also in the OptFert (−29% from Ref), HB‐0 (−37%) and R‐100 (−63%) scenarios (Table S6). In the OptFert scenario, this is driven by lower mineral N utilization and consequently lower N_2_O emissions (−23%) and emissions from fertilizer production (−38%). In the other two scenarios, the key driver is that the displacement of fossil fuel emissions is greater (118% and 201% higher than Ref respectively) than the emission equivalents from SOC losses (52% and 88% higher than Ref). Comparing the R‐0 scenario to Ref, we observe that even though SOC losses are lower (−44%), there is no emission reduction from fossil fuel displacement, therefore resulting in 32% higher total emissions. In the HB‐400 and FullCov scenarios, there is a mild reduction in GHG emissions from Ref (−4% and −5% respectively). The former is mainly due to higher displaced fossil fuel emissions and the latter due to lower SOC losses.

## 4 DISCUSSION

### 4.1 Methodological approach

Our methodology, based on the combination of stakeholder interviews with spatiotemporally explicit modelling, is a well‐suited approach towards the identification of site‐specific solutions. It allows taking into account local stakeholder perceptions on crop residue exploitation and designing agricultural management measures accordingly. Assessing these measures via modelling addresses the spatial variability of agricultural production and the complexity of agroecosystems. Only few studies have applied such a comprehensive high resolution assessment to evaluate agricultural management practices for sustainable crop residue exploitation. Despite the greater insight into the large spatial variability of measures, there are still some methodological issues which require attention and further research.

In our study, we assume static land use and do not address changes in prices and demands for food and residues for different purposes, extensive diversity in rotational patterns and evolution in the size of pasturelands. As emphasized in our study, land use patterns have a significant influence on our results regarding both residue potentials and environmental variables. In NRW in the last decades, we observe some competition between agricultural land versus inhabited areas and forests. Between 1975 and 2005, (a) cropland decreased from 38% to 35% of total land, (b) pasture and meadows decreased from 29% to 23%, (c) inhabited areas, including settlements, roads, etc. increased from 9% to 14% and (d) forest land increased from 23% to 26% (Goetzke, 2010). Similar changes in the future would have a limited influence on our absolute estimates over NRW, and a negligible effect on relative differences between scenarios. Regarding the use of residues for animal husbandry in NRW, we note that Weiser et al. (2014) estimate those to about 400 kt/year, which is equivalent to about 15% of our residue potential estimates (2,823 kt/year).

The emission calculation is based on a limited set of emission sources. Although these capture the most critical emission drivers related to our scenarios, they should by no means be interpreted as a complete emissions balance. Furthermore, logistic aspects associated with the density of residue production and distribution have not been considered although these would impact emissions.

Our results are based on mid‐century projections and a scenario with a mild climate signal. Global warming, later in the century, would most likely speed up the decay of SOC (EC, 2009b), leading to higher emissions and lower SOC stocks. In this case, the effect of integrated management as represented by our SI scenario may underperform with respect to SOC conservation.

Crop modelling is also permeated with uncertainties that challenge the confidence placed in the results of model‐based systems (Ogle et al., 2010). The design choices made by the modellers during model development combined with the imperfect knowledge about biophysical processes and the shortage of high‐quality experimental data result in model structural and parameter uncertainties (Post, Hattermann, Krysanova, & Suckow, 2008; Tao et al., 2018). The MONICA model has been previously tested for simulating the main crops that make up the rotations of this study (see Section 2.3.1) and the effects of agricultural management on SOC (Specka et al., 2016). Nevertheless, the C input from crop residues, the relative contribution of roots and above‐ground organs to soil organic matter (Kätterer, Bolinder, Andrén, Kirchmann, & Menichetti, 2011) and the simulation of SOC decomposition are important sources of uncertainty for the current study.

Moreover, the model does not address some of the relations between SOC and soil processes that could exacerbate the differences observed here between different residue exploitation strategies. For example, it currently does not account for the effect of soil organic matter, and consequently residue retention, on soil hydraulic properties or evaporation (Bescansa, Imaz, Virto, Enrique, & Hoogmoed, 2006) or the protection they provide against water and wind erosion (Wilhelm, Johnson, Karlen, & Lightle, 2007); factors that sustain primary production while preventing soil degradation processes (Lal, 2005). This calls for the application of multi‐model ensembles, ideally considering models that have such missing functionalities built in, to investigate the contribution of model structure, parameters and climate projections (Tao et al., 2018) towards quantifying the uncertainty that afflicts model predictions.

Exploring the sensitivity of our results to more comprehensive land use assumptions, influenced by changes in demand for different agricultural commodities, as well as to different climate scenarios, are interesting future research directions. Given the impact of soil textures and initial SOC levels on SOC decline, testing a humus balance approach that differentiates between these factors would add to the literature. Also, other means of adding C to the soils, such as biochar (Atkinson, Fitzgerald, & Hipps, 2010), could be explored. Finally, given uncertainty in the GHG reduction potential of crop residues, a systematic synthesis of the literature on these estimates would be of value.

### 4.2 Generalization of obtained results

Our estimates of residue potentials are in agreement with those in other studies on NRW. On a per hectare basis, we estimate 30–40 GJ ha^−1^ year^−1^ for most of NRW in the Ref scenario, which can decrease to less than 20 GJ/ha or increase to more than 50 GJ/ha in the SI scenario. In a case that assumes 33% of residues remaining on the field, Weiser et al. (2014) estimate residue potentials of 50–70 GJ/ha. Haase et al. (2016), in their *Base* scenario where either 40% or 80% of residues are left on the field depending on initial SOC levels, estimate potentials of 15.3 GJ ha^−1^ year^−1^ from cereal straw, with this lower level likely due to their more stringent scenario assumptions. Similar potential estimates appear to be identified in European studies depicting NRW as part of their geospatial analysis (e.g. Cintas, Berndes, Englund, Cutz, & Johnsson, 2018; Hamelin et al., 2019), although a detailed comparison is limited since these studies do not present their exact regional estimates. At the level of NRW, we estimate technical potentials of about 50 PJ/year, which is equivalent to about 3,000 kt DM residues/year. Weiser et al. (2014) estimate potentials of 2,019 kt/year and Haase et al. (2016) 3,146 kt/year.

Our results indicate that SOC continues to decline in many localities of NRW, even in the case of humus balance approaches. This is supported by a recent publication of Steinmann et al. (2016), which by sampling soils in arable sites in the Cologne‐Bonn region, found that despite humus conservation practices, SOC stocks continue to decline. Other studies also hint that the humus reproductive capacity of crop residues may be lower than assumed by current humus balance approaches (Lindorfer et al., 2014; Münch, 2008). Nevertheless, our scenario analysis indicates that the removal of residues can be compensated to some degree by modified management practices, as also pointed out by Lindorfer et al. (2014). In particular, cover crops are identified as a positive measure against SOC decline (EC, 2009a).

Greenhouse gas emissions are hard to compare across studies since the considered sources of emissions differ substantially. Regarding emissions due to SOC losses, our estimate is in average 29 g CO_2‐eq_/MJ bioethanol (yearly average between base year and 2050, see Section 2.3.4). This estimate relies heavily on local pedoclimatic conditions and the temporal horizon of a study (Sheehan et al., 2014). For example Liska et al. (2014), with a focus on the US Corn Belt, assuming a significantly shorter temporal horizon, estimate an average 48.8 g CO_2‐eq_/MJ over a 10 year period or 69.5 g CO_2‐eq_/MJ over 5 year. Our N_2_O reduction estimate (−4.7 g CO_2‐eq_/MJ bioethanol) is very close to this of the above‐mentioned study. We estimate a bioethanol production emission intensity equal to 40 g CO_2‐eq_/MJ bioethanol, close to estimates in other studies, such as for example Lindorfer et al. (2014), which estimate 34.1 g CO_2‐eq_/MJ bioethanol. However, we do note that these values, as shown by, for example, Lindorfer et al. (2014) and Spatari and MacLean (2010), remain highly variable depending on the emission sources, allocation methods, temporal horizons and conversion technologies considered.

The results of our stakeholder consultation are in line with those of Glithero et al. (2013) who identified the benefits of straw incorporation as an important reason for farmers not baling their straw and market developments (prices and market existence) as a potential incentive for expanding residue utilization for bioenergy. Timeliness of operations (i.e. delays in establishing the next crop because of baling) was another important reason mentioned in their analysis, which is not considered in our case, likely due to the small number of farmers in our stakeholder sample.

Finally, our results confirm that the availability of crop residues for bioenergy can only be analysed in a spatially explicit manner as emphasized by earlier studies (e.g. Haase et al., 2016; Lindorfer et al., 2014), given the impact of prevailing land use, management and pedoclimatic structures and the capacity of spatially explicit assessments to inform decision making prior to implementation of residue utilization strategies. For example, we find that SOC losses tend to be greater in light soils, compared to medium and heavier soils, as also shown by other studies (e.g. Bot & Benites, 2005; Drewniak, Mishra, Song, Prell, & Kotamarthi, 2015), thus indicating lower residue removal in those areas (Batidzirai et al., 2016).

## 5 CONCLUSIONS

Our study employs an integrated approach based on stakeholder interviews and spatiotemporally explicit quantitative assessment to explore opportunities and challenges for the SI of crop residue exploitation for bioenergy. The approach allows capturing the complexity of agroecosystems and addressing the spatial variability of agricultural production by tailoring management to pedoclimatic factors and land use.

Our results indicate that integrated site‐specific agricultural management, based on the combination of humus balancing, optimized fertilization and winter soil cover, can enhance the SI of crop residue exploitation. A scenario based on the combination of all three measures performs better than our reference case with respect to all investigated variables. At the level of NRW, we observe (a) 5% increase in technical residue potentials and displaced emissions from the substitution of fossil fuels by bioethanol, (b) 8% decrease in SOC losses and associated emissions, (c) 18% decrease in N_2_O emissions, (d) 37% decrease in mineral N fertilizer requirements and emissions related to their production and (e) 16% decrease in nitrate leaching. The humus balance achieves higher residue potentials. Optimized fertilization reduces the utilized mineral N and nitrate leaching. Greater soil winter cover ameliorates SOC levels. GHG emissions are reduced via four distinct channels. First, higher potentials result in greater displacement of fossil fuels and their associated emissions. Second, lower SOC losses result in greater C sequestration. Third, lower mineral N use is associated with lower N_2_O emissions and emissions resulting from the production of fertilizers. These results are spatially differentiated, with residue potentials being higher in areas where winter cereals dominate, and SOC decline and nitrate leaching being more pronounced in areas with light soils.

Despite the synergistic effects identified above, significant trade‐offs between energy potentials and soil impacts emerge under suboptimal management. As identified by stakeholder interviews and our quantitative scenario analysis, excessive residue extraction exacerbates SOC loss. This, in turn, can affect soil fertility and yields, as well as soil‐based climate change mitigation. Therefore, residue removal strategies need to be tailored to explicit policy targets for SOC conservation. Additionally, further verification of the humus balance approach as a sufficient sustainability criterion is needed, as well as a discussion on the feasibility of halting SOC decline without C additions by other means, such as biochar. Besides these agronomic and environmental concerns, the stakeholders identified that market and technological developments or competition with other residue uses are challenges that may hinder the expansion of crop residue exploitation for bioenergy in the near term.

Our study confirms that crop residue exploitation for bioenergy is subject to spatial variability and agronomic, environmental and market challenges. This calls for a combination with other measures, such as energy and residue demand management and exploitation of other renewable sources, in order to reach ambitious climate change mitigation targets. Nevertheless, in the frame of sustainable agricultural intensification, optimized site‐specific integrated agricultural management can simultaneously increase crop residue potentials for bioenergy and enhance environmental sustainability by improving soil conditions and reducing water pollution. It can, therefore, play an important role in mitigating climate change, while producing cobenefits for the environment and enhancing agricultural productivity.
