# Supplementary material for: Sustainable intensification of crop residue exploitation for bioenergy: Opportunities and challenges
Source: Glob Change Biol Bioenergy. 2019 Oct 31;12(1):71–89. doi: 10.1111/gcbb.12649 (PMC6988490; doi:10.1111/gcbb.12649)
Supplement: Supplementary file 1 [file GCBB-12-71-s001.pdf]

Supporting Information for Research Article

Sustainable intensification of crop residue exploitation for bioenergy: opportunities and challenges

Ioanna Mouratiadou[1*], Tommaso Stella[2], Thomas Gaiser[3], Birka Wicke[1], Claas Nendel[2], Frank Ewert[2,3], Floor van der Hilst[1]

[1] Copernicus Institute of Sustainable Development, Utrecht University, Princetonlaan 8a, 3584 CB Utrecht, the Netherlands
[2] Leibniz Centre for Agricultural Landscape Research (ZALF), 15374 Müncheberg, Germany
[3] Institute of Crop Science and Resource Conservation, University of Bonn, 53115 Bonn, Germany

*Corresponding author, e-mail: i.mouratiadou@uu.nl

Table of Contents

1 Information on the case-study region 2
2 Data and assumptions 4
2.1 Residue management 4
2.2 Fertilisation and cover crops 5
2.3 Emission calculations 8
3 Simulated and observed SOC dynamics 9
4 Additional results 10

1 Information on the case-study region

NRW is a highly productive and intensively cultivated region located in the central western part of Germany. It is the most populous and one of the most productive German states accounting for more than 20% of Germany's Gross Domestic Product (Staatskanzlei des Landes Nordrhein-Westfalen, 2016).

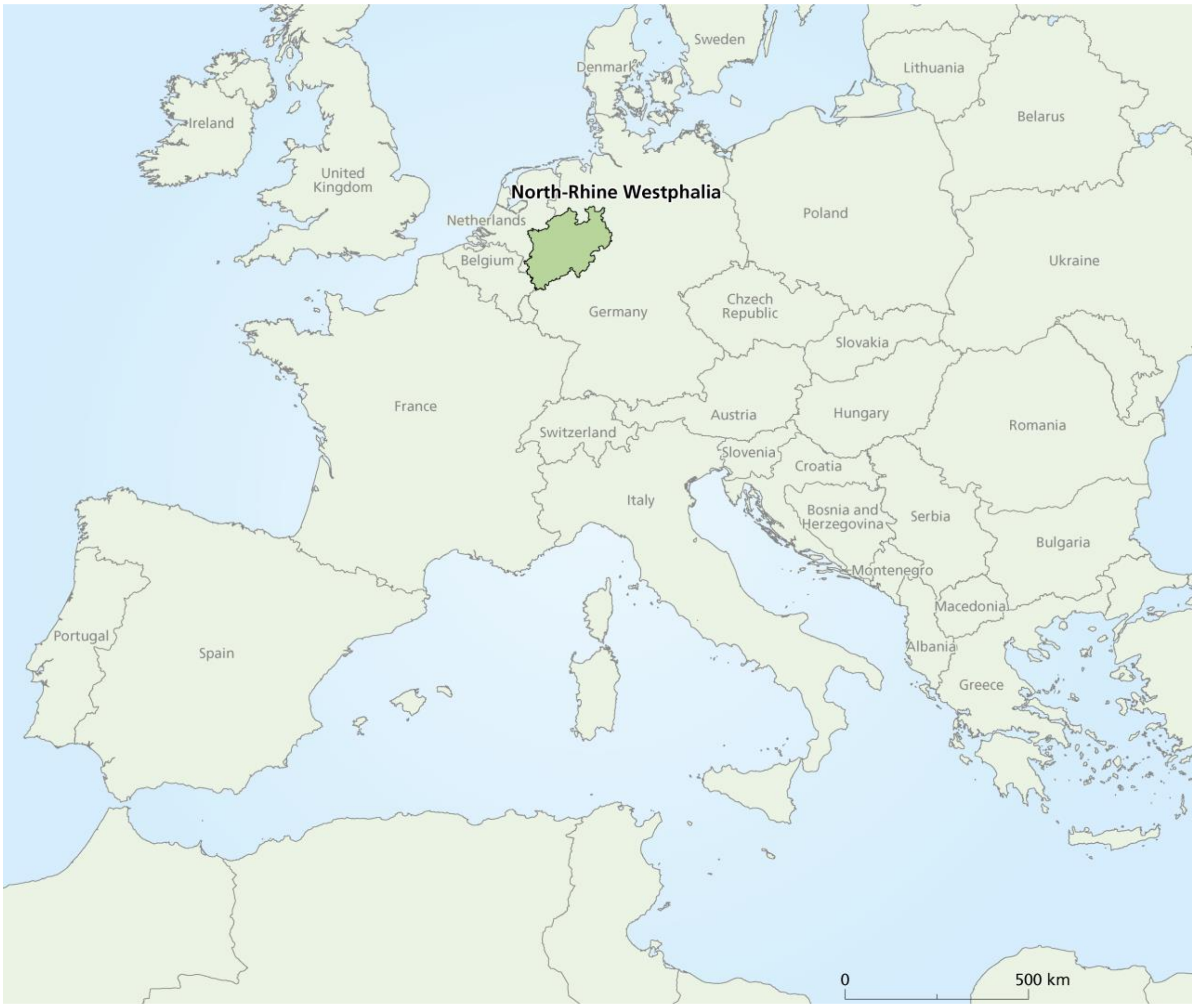

Figure S1. Location of NRW.

In NRW, annual rainfall varies between 600 mm in the lowland (north and west NRW) and 1,400 mm in the highlands (northeast and south NRW) and average temperature ranges between 5-10 °C (LWK NRW, 2014). The northwestern part of NRW (Munster and parts of Detmold and Dusseldorf) is mainly characterized by less fertile light soils. Soils are most fertile on the north eastern and southern plains.

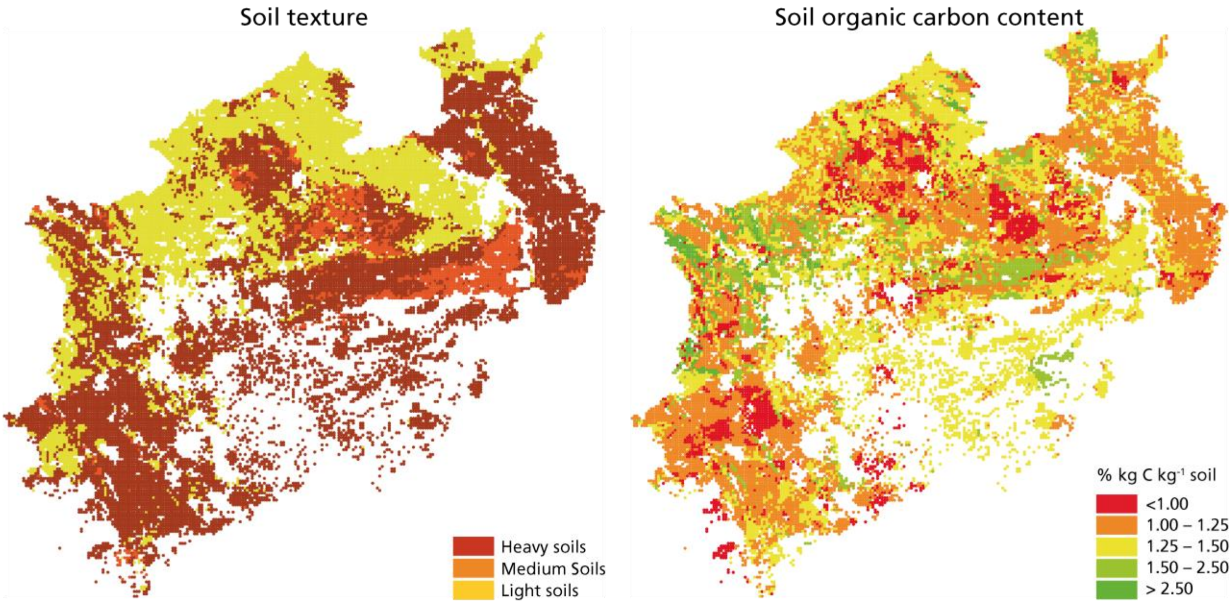

Figure S2. Texture (left) and organic carbon content (right) of soils in NRW. For the soil texture map we aggregated the 31 soil texture classes from the German soil-classification system (AG Boden, 2005) into light (sandy), medium (silty and loamy) and heavy (clayey) soils. The soil organic carbon (SOC) map shows baseyear (2000-2004) SOC levels simulated by the MONICA model after model calibration.

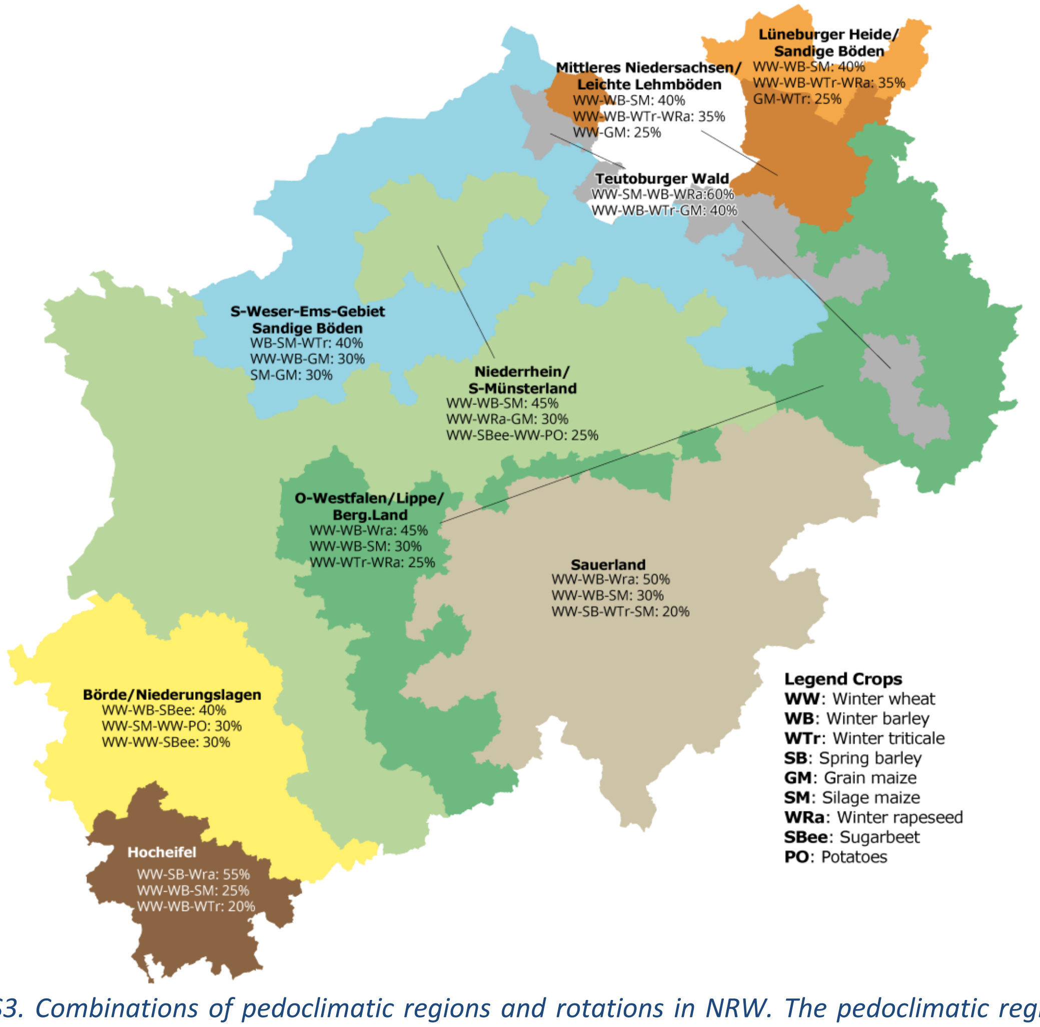

Figure S3. Combinations of pedoclimatic regions and rotations in NRW. The pedoclimatic regions are retrieved from Roßberg et al (2007)and the percentage of rotations per pedoclimatic region from Burkhardt and Gaiser (2010) based on statistical reports on cropland shares of major crops in 2007.

2 Data and assumptions

2.1 Residue management

The equation used for the agricultural management scenarios based on the humus balance approach is shown below:

$$Bal = HumDem_c - RES_{i,c,y}\ x\ HumRes_c - Org_i x\ HumOrg - CC_i\ x\ HumCC - HUMCO_{i,y'} \quad (1.)$$

Where, **c**: crop; **y:** year; **y'**: previous year; **Bal**: scenario-specific required humus balance level (H_{eq} ha^{-1} yr^{-1}); **HumDem** $_c$: humus demand factor per crop (H_{eq} ha^{-1} yr^{-1}); **RES$_{i,c}$**: residues remaining on the field (t dry matter (DM) ha^{-1} yr^{-1}); **HumRes$_c$**: humus supply factor for residues (H_{eq} t^{-1} DM); **Org$_i$**: amount of applied organic fertiliser (t ha^{-1} yr^{-1}); **HumOrg**: humus supply factor for organic fertiliser (H_{eq} t^{-1} DM); **CC$_i$**: cover crop biomass (t ha^{-1} yr^{-1}); **HumCC**: humus supply factor for cover crops (H_{eq} t^{-1} DM); **HUMCO$_i$**: humus balance in previous cultivation period (H_{eq} ha^{-1} yr^{-1}).

Table S1. Humus balance data and assumptions

Parameter or Variable	Source	Data and assumptions
**Bal**	LWK NRW (2015) and own assumption	0 or 400 H_{eq} ha^{-1} yr^{-1} depending on scenario
**HumDem**	LWK NRW (2015)	Cereals (except maize) and winter rapeseed: 280 H_{eq} ha^{-1} yr^{-1}<br>Silage and grain maize: 760 H_{eq} ha^{-1} yr^{-1}<br>Sugar beet and potatoes: 560 H_{eq} ha^{-1} yr^{-1}<br>Cover crops: 140 H_{eq} ha^{-1} yr^{-1}
**RES**	MONICA	Residue yields are simulated by the model; the share of residues remaining on the field are determined at harvest by solving Eq. 1.
**HumRes**	LWK NRW (2015)	116 H_{eq} t^{-1} DM
**Org**	LWK NRW (2014)	Organic fertilization is based on organic N budget estimated at district level. See Figure S4.
**HumOrg**	LWK NRW (2015)	100 H_{eq} t^{-1} DM; we assume all organic fertilizers are in the form of slurry.
**CC**	MONICA	Relevant only for summer crops; yields of cover crops are simulated by the model.
**HumCC**	LWK NRW (2015)	80 H_{eq} t^{-1} DM
**HUMCO**	MONICA	Humus balance carried over from the previous cropping season. A deficit is established when the amount of residues produced by a crop plus the organic fertilization are not sufficient to meet the target balance (Bal). A surplus may exist for crops returning all the residues to the soil (e.g., winter rapeseed).

2.2 Fertilisation and cover crops

All fertilization strategies assume a crop-specific average N requirement to attain optimal growth and yield (target N in Table S2) derived from LWK NRW (2016). Part of this N demand is covered by the mineralization of soil organic matter and organic fertilizers. The remainder (i.e. target N – soil N supply) is applied as mineral N. The mineral N target is split into different fertilization events, with timing and relative dosage determined from recommendations (LWK NRW, 2016) (Table S2).

Table S2. Parameters for the characterization of mineral N fertilization.

Crop	Target N (kg ha^{-1})	Dosage (fraction)			Timing (fixed date or phenological stage)			Target soil depth (cm) **		
		F1	F2	F3	F1	F2	F3	F1	F2	F3
**Silage maize***	190	1	-	-	31/05	-	-	60	-	-
**Grain maize***	190	1	-	-	31/05	-	-	60	-	-
**Spring barley**	150	0.67	0.33	-	sowing	double ridge	-	60	60	
**Winter-Triticale**	190	0.37	0.32	0.31	15/03	double ridge	flowering	90	90	90
**Winter rape**	180	0.55	0.45	-	01/03	double ridge	-	90	90	-
**Sugarbeet**	180	0.67	0.33	-	sowing	emergence	-	90	90	-
**Winter wheat**	200	0.35	0.33	0.32	15/03	double ridge	flowering	90	90	90
**Winter barley**	180	0.39	0.31	0.3	15/03	double ridge	flowering	90	90	90
**Potato**	160	1	-	-	sowing	-	-	60	-	-

Source: Landwirtschaftskammer Nordrhein-Westfalen (2016) and own elaboration as described in text. Timing is either defined as a fixed date or as a phenological stage. In the latter case, fertilization is fired when the MONICA model hits a determined development stage (sowing, emergence, double ridge, or flowering).
* Additional 20 kg N ha^{-1} are supplied at sowing.
** Used only for Fert and SI scenarios.

The way soil N supply is estimated is scenario-dependent. For the *rule-based* option, soil N supply is provided by monitoring stations covering the study area (1996-2007), which record the N availability in soil at the begin of vegetative growth for different combinations of crop sequences and soil type (Table S3) (LWK NRW, 2018). The estimated soil supply is then adjusted by the effects of cover crops and organic N management following the official recommendations (LWK NRW, 2016). In specific, the presence of a cover crop prior to the main crop increases the supply by 20 kg N ha^{-1} and each livestock unit (corresponding to 100 kg N from organic fertilizer) increases it by 10 kg N ha^{-1}.

For the *optimised* option, we assume farmers are able to measure soil N supply at each fertilization event, and apply the precise amount of mineral N required to meet the target N in the rooted zone. The target N is partitioned among different events according to the same dosage rules used for the rule-based option (Table S2). Applied mineral N is adjusted to compensate for the difference between the event-specific target N (i.e. crop target N x dosage coefficient) and the mineral N content simulated in the rooted zone the day of fertilisation.

Regarding cover crop assumptions, we use mustard, as it is one of the most common ones in NRW. Sowing and harvest dates of the cover crop are set dynamically during the simulation (after the harvest of the previous main crop and no later than September 20th) in order to minimize the fallow period. Harvest of the cover crop occurs ten days before the date of sowing of the following main crop.

Table S3. Average soil N supply (kg N ha^{-1}) for the crop sequences identified in NRW

**Current crop**	**Soil type**	**Previous crop**								
		Winter wheat	Winter triticale	Winter barley	**Silage maize**	Grain maize	Winter rape	Spring barley	Sugar-beet	Po-tato
**Winter wheat**	**light**	36	36	-	**33**	33	49	-	37	49
	**med**	42	42	-	**46**	46	49	-	43	49
	**heavy**	44	44	-	**47**	47	51	-	46	52
**Winter triticale**	**light**	30	-	30	**24**	29	-	30	-	-
	**med**	39	-	39	**38**	38	-	39	-	-
	**heavy**	42	-	42	**39**	39	-	42	-	-
**Winter barley**	**light**	24	24	-	**24**	-	-	-	-	-
	**med**	34	34	-	**39**	-	-	-	-	-
	**heavy**	35	35	-	**42**	-	-	-	-	-
**Silage maize**	**light**	22	22	22	**-**	27	-	-	-	-
	**med**	30	30	30	**-**	36	-	-	-	-
	**heavy**	32	32	32	**-**	30	-	-	-	-
**Grain maize**	**light**	22	28	28	**27**	-	26	-	-	-
	**med**	30	37	37	**36**	-	39	-	-	-
	**heavy**	32	31	31	**30**	-	34	-	-	-
**Winter rape**	**light**	23	23	23	**-**	-	-	23	-	-
	**med**	28	28	28	**-**	-	-	28	-	-
	**heavy**	28	28	28	**-**	-	-	28	-	-
**Spring barley**	**light**	25	-	-	**-**	-	-	-	-	-
	**med**	35	-	-	**-**	-	-	-	-	-
	**heavy**	35	-	-	**-**	-	-	-	-	-
**Sugar-beet**	**light**	31	-	**31**	**-**	-	-	-	-	-
	**med**	40	-	**40**	**-**	-	-	-	-	-
	**heavy**	40	-	**40**	**-**	-	-	-	-	-
**Potato**	**light**	22	-	**-**	**-**	-	-	-	-	-
	**med**	30	-	-	-	-	-	-	-	-
	**heavy**	32	-	-	-	-	-	-	-	-

For the main crops, typical sowing and harvest dates were identified for each pedoclimatic region (Table S4). The former were used to set the fixed sowing date of each crop, whereas the latter allowed defining a latest date of harvest (i.e. 15 days after typical harvest date). Harvest is triggered either at maturity, if this is reached before the latest date, or at the latest date defined.

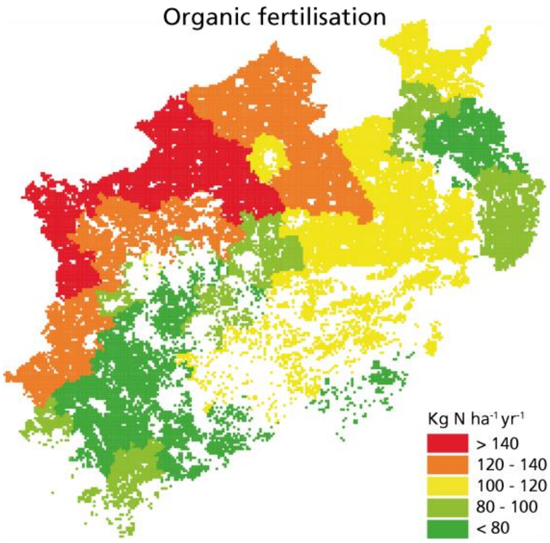

Figure S4. Organic fertilisation levels in NRW. The map is based on the organic N balance of farms estimated at district level (LWK NRW, 2014, Table 27, p. 52).

Table S4. Typical sowing and harvest dates per crop.

Redo-climatic zone	Dates	Winter wheat	Winter barley	Grain maize	Silage maize	Winter rapeseed	Sugar-beet	Spring barley	Potato
**129**	**Sowing**	04/10	17/09	NA	25/04	11/09	NA	31/03	NA
	**Harvest**	15/08	17/07	NA	26/09	15/07	NA	15/07	NA
**134**	**Sowing**	04/10	17/09	NA	25/04	01/09	NA	31/03	NA
	**Harvest**	10/08	17/07	NA	26/09	15/07	NA	15/07	NA
**141**	**Sowing**	31/10	27/09	28/04	25/04	15/09	01/04	NA	NA
	**Harvest**	20/07	04/07	20/10	15/09	01/07	05/11	NA	NA
**142**	**Sowing**	16/10	27/09	28/04	25/04	15/09	01/04	NA	NA
	**Harvest**	20/07	04/07	20/10	15/09	01/07	05/11	NA	NA
**143**	**Sowing**	04/10	17/09	NA	25/04	15/09	NA	NA	NA
	**Harvest**	10/08	10/07	NA	15/09	15/07	NA	NA	NA
**146**	**Sowing**	NA	NA	NA	NA	NA	NA	NA	NA
	**Harvest**	NA	NA	NA	NA	NA	NA	NA	NA
**147**	**Sowing**	NA	NA	NA	NA	NA	NA	NA	NA
	**Harvest**	NA	NA	NA	NA	NA	NA	NA	NA
**148**	**Sowing**	16/10	27/09	28/04	25/04	15/09	01/04	NA	NA
	**Harvest**	20/07	04/07	01/10	15/09	01/07	05/11	NA	NA
**191**	**Sowing**	04/10	17/09	NA	25/04	01/09	NA	NA	NA
	**Harvest**	10/08	17/07	15/10	20/09	22/07	NA	NA	NA
**German average (1980-2010)**	**Sowing**	09/10	20/09	29/04	29/04	26/08	10/04	31/03	24/04
	**Harvest**	10/08	18/07	28/09	28/09	26/07	07/10	09/08	16/09

Source: Bundessortenamt (2000). For each crop, missing dates (NA) were replaced by the average of crop operations in NRW pedoclimatic regions for which the data is available. For potato, we used the German average as retrieved from the German weather service (DWD) phenological database (DWD Climate Data Center, 2018). Winter triticale dates are assumed as the average of winter wheat and winter rye. For the codes of the pedoclimatic regions see Figure S2.

2.3 Emission calculations

Table S5. N concentration of above-ground residues.

Crop	N concentration (kg N kg^{-1} DM)
**Winter wheat**	0.006
**Winter triticale**	0.006
**Winter barley**	0.007
**Spring barley**	0.007
**Silage maize**	0.006
**Grain maize**	0.006
**Winter rape**	0.015
**Sugarbeet**	0.016
**Potato**	0.019
**Cover crop**	0.015

Source: IPCC (2006)
Note: for winter rape and the cover crop (mustard) we assumed the value for non-N fixing forages.

3 Simulated and observed SOC dynamics

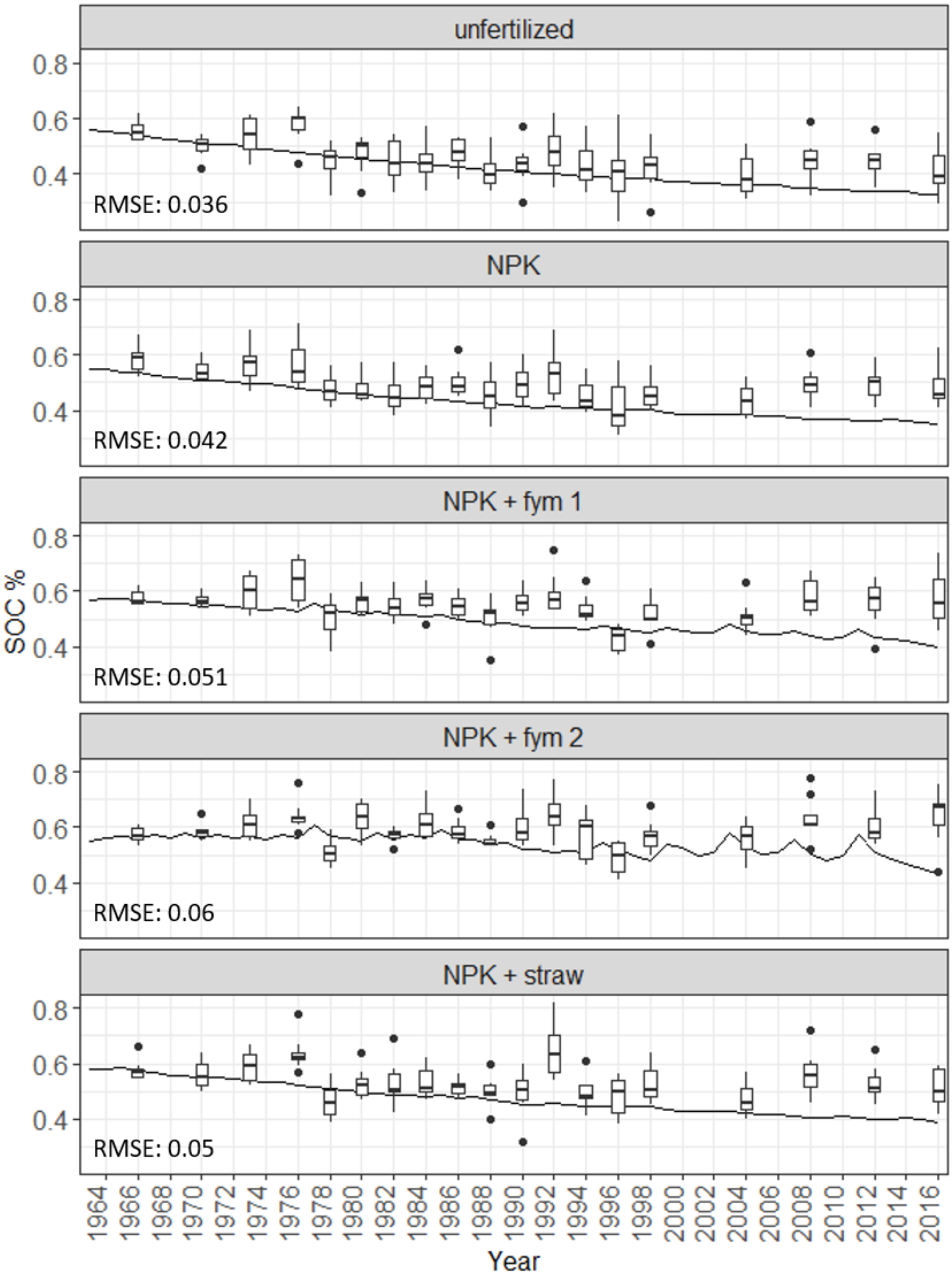

Figure S5. Simulated (continuous lines) and observed (boxplots) SOC dynamics from long- term field experiments carried out in Müncheberg, Germany. Details about the treatments are reported by Rogasik et al. (2004); NPK = mineral fertilization, fym = farmyard manure. For Fym 1, fym 2 and straw treatments respectively 1.2, 3.2 and 2.0 t DM ha^{-1} of organic manure were applied to root crops (Stella et al., 2019).

4 Additional results

Table S6. Level of technical potential and environmental impacts at the level of NRW per agricultural management scenario in 2050. For each variable, we present absolute values and per cent change to the Ref scenario. The estimation of the variables is based on the equations shown in Section 'Estimation of technical residue potentials and GHG emissions'.

	Technical potential		ΔSOC		Nitrate leaching		GHG emissions	
	PJ yr^{-1}	%	kt C yr^{-1}	%	kt N yr^{-1}	%	kt CO_{2-eq} yr^{-1}	%
Ref	48	-	-281	-	108	-	2623	-
SI	50	5	-257	-8	91	-16	1752	-33
HB-400	52	9	-283	1	109	1	2518	-4
OptFert	47	-2	-290	3	91	-16	1865	-29
FullCov	48	0	-242	-14	102	-5	2491	-5
R-0	0	-100	-156	-44	121	12	3465	32
HB-0	105	118	-425	52	97	-10	1644	-37
R-100	145	201	-527	88	90	-16	958	-63

Table S7. GHG emissions from the displacement of fossil fuels by bioethanol, changes in SOC, N_2O emissions, and emissions for the production of fertilisers in 2050 (10^3 tonnes CO_{2-eq} yr^{-1}). For each variable, we present absolute values and per cent change to the Ref scenario.

	Displaced fossil fuel emissions		Emissions equivalents of SOC changes		N_2O emissions		Fertiliser production emissions	
	Absol.	%	Absol.	%	Abs.	%	Abs.	%
Ref	-1225	0	1029	0	1681	0	1139	0
SI	-1284	5	942	-8	1379	-18	715	-37
HB-400	-1338	9	1038	1	1679	0	1139	0
OptFert	-1195	-2	1062	3	1288	-23	710	-38
FullCov	-1224	0	888	-14	1729	3	1098	-4
R-0	0	-100	572	-44	1754	4	1139	0
HB-0	-2672	118	1558	52	1619	-4	1139	0
R-100	-3691	201	1931	88	1578	-6	1139	0

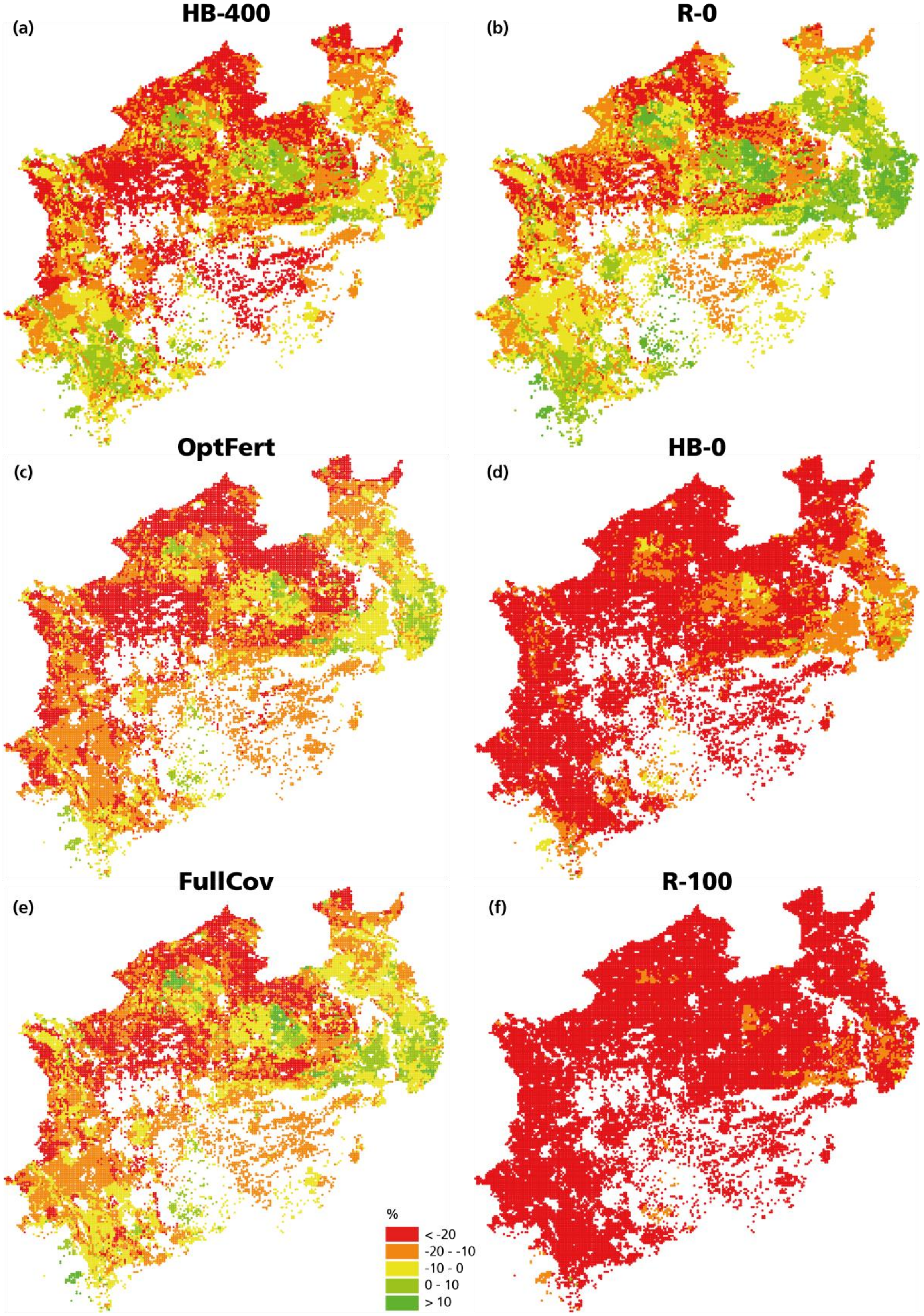

Figure S6. Spatially explicit ΔSOC in topsoil between baseyear and 2050 for different agricultural management scenarios.

References

AG Boden. (2005). *Bodenkundliche Kartieranleitung (KA5). 5th revised edition.* Stuttgart: Bundesanstalt für Geowissenschaften und Rohstoffe, E. Schweitzerbart'sche Verlagsbuchhandlung.

Bundessortenamt. (2000). *Planungs-, Informations- und Auswertungssystem für Feldversuche (PIAF).* Retrieved from https://www.bundessortenamt.de/internet30/index.php?id=155

Burkhardt, J., & Gaiser, T. (2010). *Modellierung der Folgen des Klimawandels auf die Pflanzenproduktion in Nordrhein-Westfalen.* Ministerium für Umwelt und Naturschutz, Landwirtschft und Verbraucherschutz des landes Nordrhein-Westfalen.

DWD Climate Data Center. (2018). *Phenological observations of crops from sowing to harvest (annual reporters, historical), Version v005*.

IPCC. (2006). Chapter 11. N2O emissions from managed soils, and CO2 emissions from lime and urea application. In *2006 IPCC Guidelines for National Greenhouse Gas Inventories* (pp. 11.1-11.54). Retrieved from https://www.ipcc-nggip.iges.or.jp/public/2006gl/pdf/4_Volume4/V4_11_Ch11_N2O&CO2.pdf

LWK NRW. (2014). *Nährstoffbericht NRW 2014*. Landwirtschaftskammer Nordrhein-Westfalen.

LWK NRW. (2015). *Humus und Bodenfruchtbarkeit*. Landwirtschaftskammer Nordrhein-Westfalen.

LWK NRW. (2016). *Ratgeber Pflanzenbau und Pflanzenschutz.* Münster, Germany: Landwirtschaftskammer Nordrhein-Westfalen.

LWK NRW. (2018). *Durchschnittliche Nmin-Richtwerte—5jähriges Mittel*. Retrieved from Chamber of Agriculture North Rhine-Westphalia (LWK NRW) website: https://www.landwirtschaftskammer.de/landwirtschaft/ackerbau/pdf/nmin-richtwerte-5-j-mittel.pdf

Roßberg, D., Michel, V., Graf, R., & Neukampf, R. (2007). Definition von Boden-Klima-Räumen für die Bundesrepublik Deutschland. *Nachrichtenblatt Deutscher Pflanzenschutzdienst*, *59*(7), 155–161.

Staatskanzlei des Landes Nordrhein-Westfalen. (2016). NRW entdecken. Retrieved 9 September 2016, from WIR IN NRW DAS LANDESPORTAL website: https://www.land.nrw/de/land-und-leute/nrw-entdecken

Stella, T., Mouratiadou, I., Gaiser, T., Berg-Mohnicke, M., Wallor, E., Ewert, F., & Nendel, C. (2019). Estimating the contribution of crop residues to soil organic carbon conservation. *Environmental Research Letters*. Retrieved from http://iopscience.iop.org/10.1088/1748-9326/ab395c
